# Personalized Accelerated rTMS to Manage Cognitive and Depression Symptoms in Alzheimer's Disease

**DOI:** 10.1002/alz70861_109022

**Published:** 2025-12-23

**Authors:** Danielle D DeSouza, Mylea Charvat, David Carreon

**Affiliations:** ^1^ Acacia Clinics, Sunnyvale, CA USA

## Abstract

**Background:**

Repetitive brain stimulation (rTMS) has been FDA‐cleared for major depressive disorder (MDD) since 2008 with recent work focusing on personalized treatments. In one approach, resting‐state functional MRI (fMRI) has been used to increase the effectiveness of TMS in MDD by identifying personalized targets. In addition, rTMS has shown promise as a safe and effective intervention for improving cognition in individuals with Alzheimer's disease (AD); however, previous clinical trials often excluded participants with MDD or other psychiatric comorbidities. Given the co‐occurrence of AD and MDD is common, with pooled prevalence estimates of around 38%, there is a critical need to investigate personalized therapeutic strategies that address both conditions.

**Methods:**

We describe the case of an 88‐year‐old woman with AD presenting to our outpatient psychiatry clinic with complaints of depression and anxiety. Her medical history noted mood and cognitive decline starting five and two years prior to presentation, respectively. At baseline, the patient had severe cognitive impairment with a Montreal Cognitive Assessment (MoCA) score of 8. We delivered an accelerated iTBS protocol (10 sessions/day, 1,800 pulses/session, over five days) targeted a left dorsolateral prefrontal cortex site anti‐correlated with the sgACC, identified by resting‐state fMRI. Due to residual anxiety and insomnia, a second accelerated course (26 sessions over five days) targeted a right prefrontal site anti‐correlated with the ventral striatum. Regular follow‐ups were scheduled to track status for 14 months.

**Results:**

Post‐treatment mood symptoms were assessed by Clinical Global Impressions–Improvement (CGI‐I) to be a score of 2, sustained for 14 months. Post‐treatment MoCA score increased to 16. Collateral data from the patient’s son and medical decisionmaker noted remarkable improvements in both mood and cognition, “it's like she's my mom again.”

**Conclusions:**

This case supports the feasibility of using personalized resting‐state fMRI‐guided rTMS protocols to manage symptoms in individuals with AD with psychiatric comorbidities, including MDD and anxiety. Future research on larger samples is warranted to evaluate the efficacy of this approach and disentangle the complex interplay of symptoms related to each condition.

